# Validity and reproducibility of food photographic estimation for evaluating meals in evacuation shelters

**DOI:** 10.3934/publichealth.2023013

**Published:** 2023-03-07

**Authors:** Haruka Kobayashi, Noriko Sudo, Nobuyo Tsuboyama-Kasaoka, Ikuko Shimada, Keiichi Sato

**Affiliations:** 1 Ochanomizu University, Tokyo, Japan; 2 National Institutes of Biomedical Innovation, Health and Nutrition, Osaka, Japan; 3 University of Kochi, Kochi, Japan; 4 Senshu University, Kanagawa, Japan

**Keywords:** food photograph, validity, reproducibility, portion size, evacuation shelter

## Abstract

**Objective:**

The primary goal was to evaluate the validity of food photographic estimation for nutritional assessment compared with weighed food record (WFR).

**Methods:**

We evaluated the validity and reproducibility of photographic estimation of foods provided in evacuation shelters. We analyzed 35 meals served at 12 shelters in Kumamoto Prefecture in Japan, affected by a heavy rain disaster in 2020. In this context, we compared 21 senior students' portion size estimation by food photographs to WFR. In addition, we assigned five meals for each of the 21 senior students, and the same meal photograph was estimated by three students to test reproducibility.

**Results:**

No statistically significant difference was detected between the two methods regarding energy, the total grams of meal, the protein, and vitamins B_1_, B_2_, and C, except for salt. In addition, the students who never self-cooked underestimated the total grams.

**Conclusion:**

Food photographic estimation could simplify the nutritional assessment in evacuation shelters. However, unclear photographs and food items served by weight could weaken the estimation accuracy. According to previous studies and the applied postestimation questionnaire, photographs taken from specified angles and reference food photobooks for portion size estimation may improve accuracy.

## 1. Introduction

Recently, the number of natural disasters such as earthquakes and torrential rains has been increasing in Japan [1]. In comparison with other developed countries, Japan has higher risk for natural disasters [2]. In affected local governments, dietary assessments in evacuation shelters provide a basis to secure food and maintain the health of the evacuees [3]. In this context, following the heavy rain disaster that happened in Kumamoto in July 2020, the Kumamoto Prefectural Government asked local and dispatched dietitians to collect weighed food record (WFR) with food photographs as supplementary information. However, WFR was time consuming for the dietitians even though it provides the most accurate dietary information [4]. A simpler and time- and manpower-saving method would be desirable for dietary assessment in emergent cases.

The validity and reproducibility of food photographic estimation have been evaluated in laboratory, cafeteria and free-living settings targeting all range of ages from infants to the elderly, using digital cameras at first, back in the 1990s, then more into advanced equipment, such as personal digital assistants and smartphones [5].

Contrary to free-living settings where a wide variety of food items are available [6], in evacuation shelters, the same meals are uniformly provided, and the menu is static and repeated every day [7]. These features in shelter meals might facilitate dietitians' portion size estimation due to the limited variety of food photographs.

This study evaluated the validity and reproducibility of food photographic estimation of shelter meals via WFR, collected at evacuation shelters in Kumamoto Prefecture in 2020. Whenever validity and reproducibility are ensured, anyone even without expertise can take food photographs and assist onsite dietitians in having more time to provide individual nutritional guidance for evacuees who need special care.

## 2. Materials and methods

### 2.1. Weighed food record with food photographs

After two weeks of heavy rain in July 2020, WFR was conducted to assess meals in evacuation shelters as a part of public services provided by the Kumamoto Prefectural Government in Japan. Therefore, statistical sampling techniques were not employed. The affected municipal governments selected three municipalities with numerous evacuees. At 12 shelters in the three municipalities, dietitians recorded all meals served daily for 2 or 3 consecutive days and collected data from 96 meals in total.

Food consumption data was not collected because of the following two reasons. Firstly, it was difficult to assess individual consumption in chaotic emergency shelters. It was considered unethical to collect dietary intake data from a significant number of evacuees who needed a rest to recover from substantial physical and psychological damages caused by the disaster. Secondly, in food service facilities for mass feeding such as schools and hospitals, nutrition management and evaluation by health authorities was based on what meals they served rather than how much individual consumers ate.

Dietitians at each shelter filled WFR following the form directions; they weighed each cooked food dividing it into its ingredients using digital cooking scales. For example, a rice ball was weighed after dividing it into rice, fillings and laver. Noting that, condiments were recorded for only 9 out of 96 meals. Dietitians placed meals on a sheet of A4 paper (ISO 216:2007) and took photographs from a straight-above view. Since standard measurement tools such as a ruler were not necessarily available in shelters, A4 paper was used as a standard scale because of its definite shape and availability.

### 2.2. Nutrition calculation

We calculated the energy, proteins, vitamins B_1_, B_2_, and C, and salt contents in the 96 meals. We selected energy, protein, and the three water soluble vitamins since Japan's Ministry of Health, Labour and Welfare set “Nutritional Reference Values to be used as Near-term Targets for Planning and Assessment of Meal Provision in Evacuation Shelters” for them because they are important in times of disaster [8]. The three micronutrients were selected since they have a short storage period in the body and their deficiencies were common in shelters. On the other hand, some minerals like calcium and iron and vitamin B_12_ have low priority within three months after disaster, and therefore, their values were not set for this period. We used salt instead of sodium for practical purpose since the Nutritional Reference Values, Dietary Reference Intakes for Japanese, the Standard Food Composition Tables [9], nutrition facts, and national health promotion plans in Japan consistently use salt.

Nutrition calculation was conducted by Excel add-in software (Excel Eiyo Plus; KENPAKUSHA Co., Ltd.), which used the newest 8^th^ edition of the Standard Food Composition Tables in Japan [9]. Regarding the meals for which the weight of condiments was not recorded, we used the food composition tables for cooked and processed foods or the supplemental recipe data in Excel Eiyo Plus, to determine the condiments weights. If the typical standard recipe of the meal could not be found in either method, we excluded the meal from the analysis. Whenever energy and nutrient contents were shown on the food label or manufacturer's website, these values were used for the analyses instead of the calculated ones.

### 2.3. Selection of food photograph

**Figure 1.**
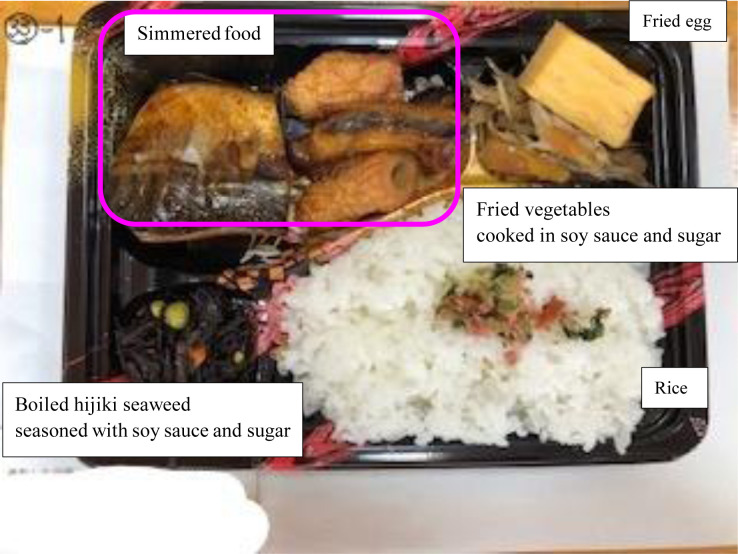
A food photograph of boxed meal. In estimation, foods in the photographs were not labelled for the participants. Due to its blurriness, energy, vitamins B_1_ and B_2_, and salt estimations from this photograph were classified into opposite quartiles of those calculated by weighed food record.

Seven meals were not photographed in error and food photos were available for 89 out of 96 meals. These 89 photos were screened according to four exclusion criteria: 1) photos with incomplete WFR (n = 21), 2) duplication of the same food (n = 13), 3) foods not listed in the food composition tables or Excel Eiyo Plus (n = 19), 4) beverage only (n = 1). Therefore, a total of 54 photos were excluded, and the remaining 35 photos were used for the analyses. Out of the 35 meals, 18 were food aid, 13 boxed meals and 4 were hot meal service. Figure 1 shows an example of boxed meal. The 35 meals consisted of 196 food items in total.

### 2.4. Portion size estimation by food photograph

We sent by email a form to 35 senior students at a women's university in Tokyo, asking for their participation in this study. They had nearly completed their educational program for registered dietitian when this study was conducted.

Overall, 24 students answered the form (response rate was 74%) and 21 of them agreed to participate in the portion size estimation by food photograph. They were not provided any training of estimation for this study but they were given a written instruction on how to estimate food weights. The written instruction included the following four tasks: 1) see photographs of meals that were placed on a sheet of A4 paper, 2) describe the names of foods in the photographs since foods were not labelled, 3) list up ingredients included in the food by freely referring to any media, and 4) estimate weight of each ingredient. They were given a reward of 3060 JPY after completing all estimation and a postestimation questionnaire.

Ingredients and weights of foods included in each meal photograph were estimated by 3 students, and 105 estimation data of 35 meal photographs were collected. In this present nutritional assessment, meal photographs were taken at the evacuation shelters and sent to research institutes that remotely backed up the affected prefecture as volunteers. Similarly, our participants received meal photographs via email. After they completed the portion size estimation of five meals randomly assigned, they sent us back by email their estimations in Excel sheet. In addition, they were allowed to freely access to any information from books and online during the estimation analysis. Based on their portion size estimation record, we calculated energy and nutrients of each meal using Excel Eiyo Plus.

### 2.5. Postestimation questionnaire

We sent a postestimation questionnaire via a Google form to all participants by email and they sent back their answers to us. We asked for the frequency of cooking through a multiple-choice question (every day, 3–5 days/week, 1–2 days/week, never), in addition to an open-ended question regarding their impression toward estimation (easiness, difficulty and ideas for improvement in estimation).

### 2.6. Statistical analyses

We used total meal weight as a direct indicator to show the difference between WFR and food photographic estimation. For to what extent food photographic estimation method could assess food intake and meal composition ingredients, we used energy and nutrient intakes and each food weight that consisted of the meal. To assess the validity of food photographic estimations, we applied three statistical analyses. In the first analysis, the total grams of meals and their contents of energy and nutrients already calculated by WFR and food photograph estimation were compared by Wilcoxon signed-rank test since they were not normally distributed according to Shapiro–Wilk test. In the second analysis, the total grams of meals and their calculated contents were divided into quartiles. Joint classification was used to examine agreement between the two methods by calculating the number of meals in the same, adjacent, and extreme quartiles. In the third analysis, we compared the difference in portion size of each food item between the two means of provision: served by weight or by piece.

To assess the reproducibility of food photographic estimation, coefficients of between-estimator variation of total grams of meal were calculated. They were also grouped by meal categories using Kruskal–Wallis test.

To examine the association between participants' cooking experience and accuracy of estimation, the total grams of meals derived from the two methods (food photographic estimation and WFR) were compared between the ones who cooked and those who did not cook by Mann–Whitney U test.

All statistical analyses were performed using IBM SPSS Statistics 27, with the significance level at 0.05.

### 2.7. Ethics approval of research

To use the WFR and photographs for this study, we obtained written permission for secondary use from the Kumamoto Prefectural Government (permission notice/health promotion department no. 885) on one condition; the names of shelters and their located municipalities were not to be revealed. The study protocol was approved by the ethics committee of Ochanomizu University (2021–53), and all participants gave informed consent.

## 3. Results

### 3.1. Validity

The differences between WFR and food photographic estimation were not statistically significant for total grams of the meals and their contents in energy and nutrients except for the salt that was overestimated by food photographs (Table 1).

**Table 1.** Differences in estimations per meal between weighed food record and food photograph.

	Weighed food record	Food photographic estimation	Difference^a^

	Median [interquartile range^b^]	Median [interquartile range]	Median [interquartile range]
Total grams of meal	416.5 [239.0–579.5]	436.3 [274.3–552]	6.5 [−26.0–63.9]
Energy and nutrient contents in meal		
Energy (kcal)	585 [527–748]	619 [509–830]	2 [−70–135]
Protein (g)	19.4 [10.8–24.9]	19.6 [12.5–29.7]	1.2 [−1.6–5.5]
Vitamin B_1_ (mg)	0.21 [0.14–0.32]	0.25 [0.15–0.36]	0.02 [−0.02–0.09]
Vitamin B_2_ (mg)	0.27 [0.19–0.38]	0.3 [0.16–0.43]	0.01 [−0.04–0.12]
Vitamin C (mg)	13 [3–32]	12 [3–26]	0 [−5–5]
Salt (g)	3 [2.2, 4.0]	3.5 [2–5]	*0.5 [−0.3–1.6]

Note: N = 35 meals; ^a^Difference was defined as food photographic estimation—weighed food record; Therefore, positive difference reflects overestimation using food photographic estimation; ^b^25th–75th percentile; *p = 0.001 using Wilcoxon signed-rank test.

Table 2 shows the degree of agreement in total grams of meals between WFR and food photographic estimation. Out of the 35 meals, 22 were classified in the same quartiles, and no meal was detected in extreme quartiles. Table 3 shows the results of the same analysis for energy and nutrients. The only meal that was classified in the extreme quartiles for energy, vitamins B_1_ and B_2_ and salt was the same meal shown in Figure 1. This meal box was underestimated by food photograph since it was classified into the 4^th^ and 1^st^ quartiles by WFR and food photographic estimation, respectively. Figure 1 shows a small and coarse image size; therefore, all the three participants mistook the ingredients of the simmered food shown in its upper left.

**Table 2.**
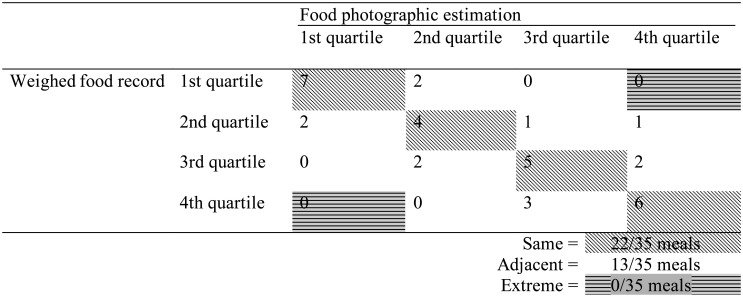
Number of meals classified into the same, adjacent, and extreme quartiles according to the total grams calculated by weighed food record and food photographic estimation.

**Table 3.** Number of meals classified into the same, adjacent, and extreme quartiles according to the contents of energy and nutrients calculated by weighed food records and food photographic estimations.

	WFR^a^ interquartile range^b^	FPE^c^ interquartile range	Same	Adjacent	Extreme
Energy (kcal)	527–748	492–782	19	15	1(53%^d^)
Protein (g)	10.8–24.9	12.6–29.9	25	10	0
Vitamin B_1_ (mg)	0.1–0.3	0.2–0.4	22	12	1(38%)
Vitamin B_2_ (mg)	0.2–0.4	0.2–0.5	22	12	1(37%)
Vitamin C (mg)	3–32	5–26	24	11	0
Salt (g)	2.2–4.0	2.4–4.9	16	18	1(52%)

Note: N = 35 meals; ^a^Weighed food record; ^b^25th–75th percentile; ^c^Food photographic estimation; ^d^FPE/WFR*100 (%).

Table 4 shows the difference in portion size of each food item between WFR and food photographic estimation. For staple foods in food aid, ratios of pictures with legible nutrition facts were 4/12 for rice ball, 0/5 for sandwich, 3/4 for jam bun and 3/4 for melon bun. The differences tended to be smaller for the foods that have more pictures of nutrition facts.

**Table 4.** Differences in portion size between weighed food record and food photographic estimation.

Food groups	Ways of provision	Food items	Frequency (times) of provision recorded in WFR^a^	Mean weight (g) recorded in WFR	Difference^b^ (%)
Staple foods	Food aid	Rice ball	12	104.9	32.9
		Sandwich	5	143.0	31.5
		Jam bun	4	120.3	11.5
		Melon bun (sweet bun covered with cookie dough)	4	100.0	20.7
	Boxed meals	Rice	12	197.1	24.3
		Rice ball	3	94.7	16.4
Main/side dishes	Served by weight	Julienned cabbage	4	12.8	58.9
		Boiled hijiki seaweed seasoned with soy sauce and sugar	4	29.4	59.1
		Fried vegetables cooked in soy sauce and sugar	4	30.4	73.4
		Cooked beans	3	20.6	68.4
		Potato salad	3	33.0	59.9
		Simmered food	3	75.1	26.5
	Served by the piece	Fried egg	13	31.5	46.2
		Fish	7	33.7	52.4
		Deep fried chicken	6	32.6	77.1
		Croquette (deep fried mashed potato)	6	58.3	48.3
		Chinese dumpling	4	16.0	40.7
		Sausage	3	10.2	41.2
		Cherry tomato	3	9.0	53.1
Japanese pickles		*Daikon* radish	6	9.6	39.7
		*Umeboshi* (salt pickled plum)	5	4.3	93.0

Note: ^a^Weighed food record; ^b^Difference (%) = means of (food photographic estimation − weighed food record)/mean weight by weighed food record × 100.

Main or side dishes served by weight tended to be more overestimated than those served by piece, but the difference between the two serving methods was not statistically significant (Mann–Whitney U test, p = 0.452). Regarding the Japanese pickles, *umeboshi* was overestimated about two times compared to other pickles.

### 3.2. Reproducibility

**Figure 2.**
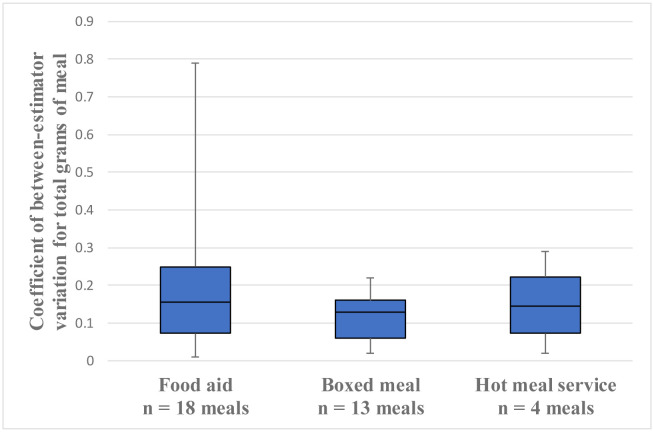
Coefficient of between-estimator variation for total grams of the same meal by categories. Each meal photograph was estimated by three participants. Kruskal–Wallis test (p = 0.613).

Figure 2 shows the coefficient of between-estimator variation for the total grams of the same meal by meal categories. Their medians [interquartile range] were as follows: 0.16 [0.07–0.25] for food aid, 0.13 [0.06–0.16] for boxed meals and 0.15 [0.07–0.22] for hot meal service. The variation was larger for food aid and hot meal service, whereas the difference between the three meal categories was not statistically significant.

### 3.3. Time required for estimation

The number of food items included in the 35 meals ranged from 1 (food aid) to 16 (boxed meal), and therefore, the time required for estimation also varied widely among participants; some took only 3 minutes to complete, and others did 1.5 h.

### 3.4. Participants' cooking experience and estimation

The responses to the postestimation questionnaire are summarized in Table 5. According to their self-reporting frequency of cooking, we divided the participants into two groups: the ones who cooked (1–7 days/week; n = 16) and those who did not (never; n = 5). When total grams of meals derived from the two methods (food photographic estimation and WFR) were compared between the two groups, the median values of the ratio [interquartile range] were as follows: 1.06 [0.98–1.26] for those who cooked and 0.93 [0.69–1.03] for those who did not cook. Participants who never cooked for themselves significantly underestimated (Mann–Whitney U test, p = 0.016). Among the former, those who cooked every day (n = 3) estimated more accurately (1.04 [0.99–1.19]).

**Table 5.** Answers to postestimation questionnaire.

Compulsory question	Frequency of self-cooking (n = 21)	Every day	3
		3–5 days/week	3
		1–2 days/week	10
		Never	5
Optional, open-ended questions	What makes estimation easy	All meals were placed on A4 paper, so it was easy to estimate their portion sizes (5).	
		Foods listed in standard tables of food composition/nutrition facts were easy to estimate (4).	
	Difficulties	I could not tell from the photographs	what condiments were used in foods (8).
			what was inside because some photographs were blurry (6).
			if it was fried (3).
			what was in the soup (1).
		It was hard to tell what was inside the box when it was covered with the clear lid and reflected the light (1).	
	Suggestions for better estimation	Take photographs from different angles (2).	
		Enlarge photographs of foods that were hard to tell what they were (1).	
		Reduce the amount of soup, and it would be easier to estimate what was in the soup (1).	
		Draw grid lines on A4 paper (1).	
		Train participants beforehand (1).	
		Prepare standard portion size model (1).	

Note: N = 21 participants; The figures in the table show the number of respondents.

### 3.5. Mistakes and oversights in estimation

Twenty-one food items were totally different from what the participants selected to be the actual ones. Especially six of them were deep fried foods with similar appearances (it was hard to tell what was inside the batter). Three of them were cases in which participants mistook red-fleshed fish with white-meat fish because the picture showed the skin-side of the fish.

Twenty-eight food items were in the picture but remained not estimated; nine were soy sauce in small container and four were cabbage used for raising bottoms. The picture of both container and cabbage was blurry because they were underneath other foods.

## 4. Discussion

### 4.1. Validity

This study first evaluated the food photographic method in evacuation shelters. The results suggested that the food photographic method could provide good estimates of the total grams (g), energy (kcal) and five nutrients compared with WFR. A comparison between the two methods demonstrated a fair agreement for total grams (g), energy (kcal), proteins (g), vitamins B_1_, B_2_ and C (mg) (Table 1). Only a significant difference between the two methods regarding salt (g) might be due to condiments and *umeboshi* (salt pickled plum). The salt content in one *umeboshi* is 7.6%–18.2% [9], and it was overestimated about two times by food photographic method. The percentage of under-estimations for both energy and salt and the percentage of under-estimations for both vitamin B_1_ and B_2_ were consistent with each other. Considering that energy and salt were taken from the meal as a whole and vitamins were taken especially from side dishes, it was likely that estimation of side dishes was particularly difficult among the food items.

The estimation of the portion sizes of the food items served by weight appeared to be more difficult than those served by piece (Table 4). Unlike foods such as Chinese dumpling and cherry tomato, whose portion size is usually shown as the number of pieces, the amount of food served by weight varied depending on how it was arranged in the box. For better estimation, one participant suggested a standard portion size model (Table 5). A previous study also reported that increasing the number of reference photographs from 3 to 8 increased estimation accuracy [10]. Furthermore, the development of a food photobook of standard portion size could aid estimation.

The main challenge was the blurriness of some photographs (Figure 1). Participants underestimated the portion sizes and could not report some food items inside the meal box especially fried foods (Table 4), which may cause underestimation of energy (kcal) and three of the nutrients; vitamins B_1_ and B_2_ (mg), and salt (g) (Table 3). Most previous studies specified the distance and angle to food in details while taking photographs [11],[12]. In fact, one of these studies reported the importance of complying the angle of photographs for better estimation of the amounts of some nutrients [13]. The angle was not fixed while taking photographs in the present study, which may have affected the lower accuracy of estimating portion sizes. When using photographs taken from a specified angle such as 45° rather than directly above, the height and the bottom area of boxes can be shown as an indicator to estimate the portion size, which may lead to more accurate estimates. In addition, when elaborating more information about food items, in particular its ingredients, participants would be less likely to mistake or miss food items in the photograph.

### 4.2. Reproducibility

Between-estimator variation was larger for food aid and hot meal service than for boxed meal (Figure 2). According to a previous study that calculated coefficients of variation for vegetable weight estimates, the values varied widely among vegetables [14]. This may be due to the estimation of soups that were not served in boxed meals. When estimating instant soups in food aid, some participants reported their dry weight before cooking, and others reported their weight cooked with hot water. For the photographs of the soups from hot meal service, it was difficult to know their content because the ingredients were submerged.

### 4.3. Participants' cooking experience and estimation

Participants who never self-cooked tended to underestimate compared to those who did, possibly because they have learned some standard portion sizes through cooking regularly. This result was consistent with previous studies [15],[16]. In food photographic estimation, accuracy might be improved if those who cook regularly are recruited through preliminary interviews.

### 4.4. Limitations and strengths

The following five points are the limitations of this study.

First, while various image processing techniques for food quality evaluation are already known [17], this study is a human-performed portion size estimation using food photograph. These methods are designed for food in normal eating conditions and may differ for food served in evacuation centers, where people store meals at room temperature and use limited heat sources. Because human-performed portion size estimation is still being conducted at disaster sites, we examined the possibility of using photographs to reduce the burden of filling out the meal survey form. This study using food photographs can be applied to meals served in shelters; however, it will be necessary to further investigate image processing techniques in the future.

Second, statistical sampling techniques were not employed since WFR was conducted as a part of public services. Therefore, the 12 shelters could not represent the whole shelters in the affected areas. The 35 meals used in this study were not necessarily the typical ones served in evacuation shelters because the dietary data were collected in one prefecture during a heavy rain disaster. Compared with recent disasters in Japan, hot meal service was limited due to hygienic concerns during the COVID-19 pandemic [18].

Third, among the 35 meals used as standards, complete records of weights of all ingredients were available for only nine meals. Many standard WFR values that were estimated based on standard recipes from the Standard Tables of Food Composition or Excel Eiyo Plus did not exactly agree with the values given on the nutrition facts labels.

Fourth, the angle and quality of photographs taken at shelters were not uniform, which may have reduced the accuracy of estimating portion sizes.

Fifth, all the participants were senior students in a dietitian training course at the same women's university. Their homogeneity in year in university, educational background, and sex could eliminate potential factors that could affect accuracy in estimation.

## 5. Conclusions

Although photographs have been used as an auxiliary tool, rather than a standalone one, combined with WFR and dietary recalls, food photographic estimations have the potential to be used as a rapid method for data collection and nutritional assessment in evacuation shelters. This study also showed some measures to improve its accuracy.

## References

[1] Small and Medium Enterprise Agency (2019). White Papers Small and Medium Enterprise in Japan.

[2] Itoh K, Kikumoto M, Shimono K (2017). A study on application of world risk index to Japan and comparison with other countries. J JSNDS.

[3] Sudo N, Shimada I, Tsuboyama-Kasaoka N (2021). Revising “nutritional reference values for feeding at evacuation shelters” according to nutrition assistance by public health dietitians based on past major natural disasters in Japan: a qualitative study. Int J Environ Res Public Health.

[4] Gibson RS (2005). Principles of nutritional assessment.

[5] Hawkins KR, Apolzan JW, Myers CA, Schoeller DA, Westerterp-Plantenga MS (2017). The assessment of food intake with digital photography. Advances in the Assessment of Dietary Intake.

[6] Saeki K, Otaki N, Kitagawa M (2020). Development and validation of nutrient estimates based on a food-photographic record in Japan. Nutr J.

[7] Itakura H, Watanabe S, Kondo K (2011). Food and Nutritional Problems Following the Great East Japan Earthquake and Tsunami disaster.

[8] Ministry of Health, Labour and Welfare (2011). Nutritional Reference Values to be used as Near-Term Targets for the Planning and Assessment of Meal Provision in Evacuation Shelters.

[9] Ministry of Education, Culture, Sports, Science and Technology (2020). Standard tables of food composition in Japan.

[10] Nelson M, Atkinson M, Darbyshire S (1994). Food photography I: the perception of food portion size from photographs. Br J Nutr.

[11] Williamson DA, Allen HR, Martin PD (2003). Comparison of digital photography to weighed and visual estimation of portion sizes. J Am Diet Assoc.

[12] Martin C, Han H, Coulon S (2008). A novel method to remotely measure food intake of free-living individuals in real time: the remote food photography method. Br J Nutr.

[13] Wang DH, Kogashiwa M, Ohta S (2002). Validity and reliability of a dietary assessment method: the application of a digital camera with a mobile phone card attachment. J Nutr Sci Vitaminol.

[14] Nishimura M, Shimada S (2018). Association between food weight measurement with the eye and dietary survey method (the photograph method). J Yasuda Women's Univ.

[15] Miyachi Y, Sasaki H (2000). A study on weighing in cooking: On visual measurement of food weights. B Sendai Shirayuri Women's Coll.

[16] Ohmori A, Harada S (2013). Actual condition of food weight in campus. B Toyama Coll.

[17] Du CJ, Sun DW (2004). Recent developments in the applications of image processing techniques for food quality evaluation. Trends Food Sci Tech.

[18] World Health Organization (2020). Disaster evacuation shelters in the context of COVID-19.

